# TWIST1 expression and clinical significance in type I endometrial cancer and premalignant lesions: A retrospective clinical study

**DOI:** 10.1097/MD.0000000000023397

**Published:** 2020-11-25

**Authors:** Junhua Shen, Qin Chen, Na Li, Xiaoxia Bai, Fenfen Wang, Baohua Li

**Affiliations:** a Department of Gynecologic Oncology; b Department of Pathology, Women's Hospital, Zhejiang University School of Medicine, Xueshi Rd no.1, Hangzhou, Zhejiang, PR China.

**Keywords:** atypical hyperplasia, endometrial cancer, premalignant lesion, risk stratification, TWIST1

## Abstract

The aim of this study was to assess the correlation of TWIST1 expression with clinical parameters and the prognosis of type I endometrial cancer (EC).

This retrospective study enrolled 345 patients. Immunohistochemical staining was performed on 55 normal endometrium (NE) samples, 27 atypical hyperplasia (AH) samples, and 263 type I EC samples. The association between TWIST1 staining and clinical characteristics and survival was evaluated by univariate and multivariate analyses.

We found significantly higher TWIST1 expression in patients with AHs and type I ECs than NEs, but there was no significant difference between TWIST1 expression in AHs and type I ECs. Aberrant TWIST1 expression was significantly associated with clinical parameters, indicating poor prognosis and shorter patient survival. Pearsons Chi-Squared test showed that high TWIST1 expression was significantly associated with a shorter disease-free survival and overall survival. More importantly, multivariate analysis showed that high TWIST1 expression, in addition to myometrial invasion, lymph vascular space invasion, and lymph node metastasis, was an independent predictor of worse DFS in patients with type I ECs.

Our findings suggest that TWIST1 might be useful in diagnosing ECs and predicting prognosis in patients with AHs and type I ECs.

## 1 Introduction

Endometrial cancer (EC) is the most common cancer of the female genital tract and the fourth most common cancer in developed countries.^[1]^ The incidence of EC is on the rise, owing to an ageing population and increased prevalence of obesity.^[2]^ ECs have been broadly classified into 2 types based upon histologic, clinical, and metabolic features. A number of recent studies have focused on type I ECs, which are most common. The progression of type I EC generally follows along the path of proliferative-to-disordered proliferative endometrium, benign hyperplasia, atypical hyperplasia (AH), and finally endometrial cancer. Sometimes the development of premalignant and malignant endometrial lesions can occur in the absence of benign hyperplasia.^[3]^

The treatment approach for type I ECs has changed in the past decade, but there is still some suspenses such as predicting AH prognosis and whether pelvic lymph node dissection is essential in EC patients. Meanwhile, stratification of patients with EC into several risk groups is currently based on post-surgical pathologic findings, including histologic subtype, tumor grade, stage, and lymphatic, vascular, and myometrial invasion, but ECs cannot be reliably classified based on histomorphologic criteria and inter-observer agreement is moderate-to-poor for histotype and tumor grade,^[4,5]^ moreover, cases of atypical hyperplasias concurrent carcinoma with deep myometrial invasion, early stage endometrial carcinomas develop recurrence and distant metastasis are not rarely seen,^[5–7]^ thus confirming that the current risk stratification systems are limited. Studies involving immunomarkers and mutational studies of single genes have been conducted in an attempt to make the sub-classification of ECs and AHs more reproducible and accurate, markers such as p53, estrogen receptor, p16, and PTEN, have been reported to predict disease outcome or prognosis of EC; however, no single marker has high sensitivity or specificity.^[8]^ Additional prognostic indicators would contribute to better detection of patients with a higher risk of relapse or death from EC.

TWIST1 is a member of the TWIST subfamily of the human bHLH protein, which is one of the key factors that regulates epithelial-mesenchymal transition (EMT).^[9,10]^ TWIST1 has been demonstrated as a novel oncogene overexpressed in diverse tumors,^[11]^ which confers more infiltrative phenotype properties to tumor cells.^[12–15]^ Some studies have demonstrated TWIST1 may be a novel molecular marker of EC severity,^[12,16]^ but it remains to be determined whether the predictive value of TWIST1 expression is sufficient to guide the clinical management of patients with type I EC, and its role play in occurrence and progression of EC.

In the present study, we examined the expression of TWIST1 protein in endometrial lesions, including normal endometrium, AH, and type I EC using immunohistochemistry. The purpose of this study was to determine the level of TWIST1 expression and clinical significance in type I EC and premalignant lesions.

## 2 Materials and methods

### 2.1 Patients and tissue specimens

This retrospective study enrolled 345 patients who were treated at the Women's Hospital at Zhejiang University School of Medicine between January 2006 and December 2013. The study population was divided into 3 groups. One group included 27 patients with AH who underwent a total hysterectomy due to fear of disease severity. The second group included 263 patients with type I EC who underwent a radical hysterectomy and bilateral salpingo-oophorectomy with pelvic lymph node dissection and who did not receive any anti-cancer therapy prior to surgery. Finally, normal endometrium (NE) tissues were collected from 55 patients who underwent hysterectomy due for a benign gynecologic disease. The present study was approved by the Institutional Review Board of the Women's Hospital (Catalogue: 20170197). Informed consent was obtained from all individual participants.

The patients with a type I EC were followed post-operatively by interview at the clinic or telephone call. Regional tumor recurrence, distant metastasis, and patient survival were recorded. Disease-free survival (DFS) and overall survival (OS) were calculated from the day of surgery until recurrence or death. The last day of follow-up was December 2016. The median duration of follow-up was 72 months (range, 4–120 months). Recurrences and deaths were recorded during the follow-up period. There were 33 recurrences (33/263 [12.55%]) and death (29/263 [11.03%]).

### 2.2 Immunohistochemical staining

Tissues were embedded in paraffin (4 μm thickness), mounted on slides, dewaxed in xylene for 15 minutes, passed through a graded aqueous/alcohol series, and rehydrated in distilled water. To recover antigen reactivity, sections were heated in 0.1 mol/L citrate buffer (pH 6) for 20 minutes in a 95°C water bath, cooled, and kept at room temperature for 30 minutes. Non-specific binding was blocked by incubating the sections for 30 minutes with dilute normal goat serum. The immunohistochemical staining was performed using the avidin-biotin peroxidase method with a SLABC kit (DAKO, Glostrup, Denmark). The slides were incubated with a rabbit polyclonal antibody against TWIST1 overnight at 37°C (1:200; Santa Cruz Biotechnology, Inc., Santa Cruz, CA, USA). Then, the sections were developed for 2 minutes with the enzyme substrate (3, 3’-diaminobenzidine chromagen; DAKO) and counterstained with Mayer's hematoxylin for 30 second. After dehydration, the tissues were cover-slipped with permount and inspected.

### 2.3 Evaluation of immunoreactivity

To evaluate TWIST1 expression, staining intensity was scored as 0 (negative), 1 (weakly positive), 2 (moderately positive), and 3 (strongly positive). The staining distribution was scored as 0 (0%–10%), 1 (11%–25%), 2 (26%–50%), 3 (51%–75%), and 4 (76%–100%) according to the percentage of the positive staining areas in relation to the total cancer areas. The sum of the staining intensity and distribution scores were used as the final staining score (0–7) for the level of TWIST1 expression. A final score <4 was considered low expression and ≥4 as high expression.

### 2.4 Statistical analysis

Statistical analyses were performed using SPSS 22.0 for Windows (SSPS, Inc., Chicago, IL, USA). Significant differences in TWIST1 expression in endometrium lesions and the relationship between TWIST1 expression and clinicopathologic characteristics were determined separately by Pearson's Chi-Squared test. Survival curves were generated by the Kaplan–Meyer method and survival rates were compared using the log-rank test. The Cox proportional hazard method was used to identify independent predictors of survival rates based on univariate and multivariate analyses. All statistical tests were two-sided and *P* values <.05 were considered statistically significant.

## 3 Result

### 3.1 Difference in TWIST1 expression in endometrium lesions

**Figure 1:**
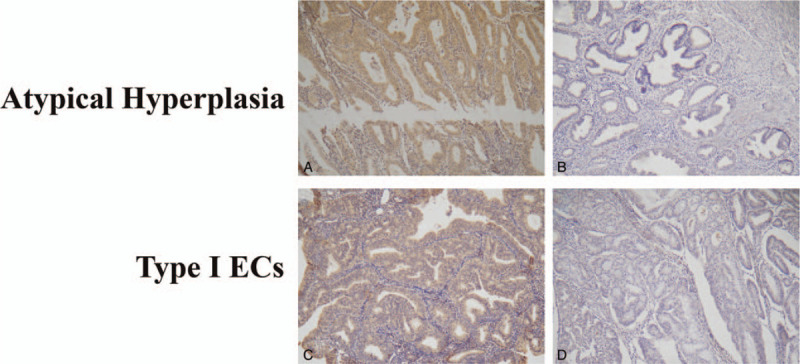
Representative immunohistochemical staining demonstrating the expression of TWIST1 in atypical endometrial hyperplasia (AH) and type I endometrial cancer (EC). TWIST1 staining was predominantly located in the cytoplasm and nucleus of AH (A) and type I EC (C). Low expression of TWIST1 was observed in AH (B) and type I EC (D).

A total of 345 endometrium tissue samples were collected to evaluate TWIST1 expression by immunohistochemistry, including 263 type I EC, 27 AH, and 55 NE. The mean patient ages were 54.58 ± 8.69 years for type I EC, 48.11 ± 8.85 years for AH, and 46.58 ± 11.41 years for NE. The mean age for the type I EC group was significantly higher than the AH (P = 2.8 E^−4^) and NE groups (P = 6.0 E^−6^), but there was no difference (*P* = .54) between the AH and NE groups. Among patients with type I EC, 183 were stage I (International Federation of Gynaecology and Obstetrics [FIGO] stages) and 80 were advanced stage (≥ II); 211 were well or moderately differentiated, and 52 were poorly differentiated; and 57 had deep myometrial invasion. Lymphovascular space invasion (LVSI) was identified in 29 cases (11.02%) and lymph node metastasis (LNM) was confirmed in 24 of 263 cases (9.13%). Immunohistochemical staining of TWIST1 expression is shown in Figure 1. The staining of TWIST1 immunoreactivity was predominantly located in the cytoplasm and nuclear of endometrium tissue samples. As shown in Table 1, 94, 94 (35.7%), (29.6%), and 3 patients (5.5%) with type I EC, AH, and NE had high TWIST1 expression, respectively. TWIST1 expression was significantly higher in patients with AH (*P* = .007) and type I EC (P = 9.0 E^−5^) than patients with NE; there was no significant difference in the AH and type I EC groups (*P* = .53).

**Table 1:** The aberrant expression of TWIST1 in 345 cases of type I endometrial cancers, atypical endometrial hyperplasia and normal endometrium.

Proteins	Expression level	Type I Endometrial cancers (n = 263)	Atypical endometrial hyperplasia (n = 27)	Normal endometrium (n = 55)
TWIST1	High	94	8	3
	Low	169	19	52

### 3.2 Correlation between TWIST1 expression with clinicopathologic characteristics in patients with type I EC

The correlation between TWIST1 expression with clinicopathologic characteristics in 263 type I EC tissues is shown in Table 2. Among 94 cases with higher TWIST1 expression, the mean patient age was 54.43 ± 9.74 years and 61 cases were postmenopausal women; however, menopausal status was not associated with TWIST1 expression (*P* = .66). Increased TWIST1 expression was significantly associated with FIGO stage (*P* = .004), deep myometrial invasion (*P* = .007), LVSI (*P* = .02), and LNM (*P* = .001), but not correlated with differentiation (*P* = .14) and tumor size (*P* = .97).

**Table 2:** The correlation between expression of TWIST1 and the clinicopathological parameters in 263 cases with type I endometrial cancers.

		TWIST1, n (%)			
Characteristic	No.	Low	High	χ^2^	*P* value
Age (years)				1.180	.277
≤ 60	195	129 (49.0)	66 (25.1)		
> 60	68	40 (15.2)	28 (10.6)		
Menopause				0.198	.656
No	97	64 (24.3)	33 (12.5)		
Yes	166	105 (39.9)	61 (23.2)		
FIGO stage				8.471	.004
<II	183	128 (48.7)	55 (20.9)		
≥II	80	41 (15.6)	39 (14.8)		
Differentiation				2.195	.138
Well/moderate	211	131 (49.8)	80 (30.4)		
Poor	52	38 (14.4)	14 (5.3)		
Myometrial invasion				7.259	.007
<1/2	206	141 (53.6)	65 (24.7)		
≥1/2	57	28 (10.6)	29 (11.0)		
Tumor size				0.002	.966
<4cm	213	137 (52.1)	76 (28.9)		
≥4cm	50	32 (12.2)	18 (6.8)		
LVSI				5.358	.021
No	234	156 (59.3)	78 (29.7)		
Yes	29	13 (4.9)	16 (6.1)		
LNM				10.997	.001
No	239	161 (61.2)	78 (29.7)		
Yes	24	8 (3.0)	16 (6.1)		

### 3.3 Survival analysis

**Figure 2:**
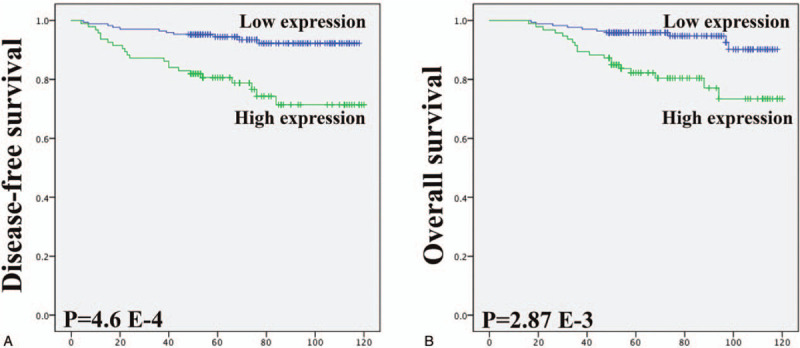
Kaplan-Meyer curves showed the association between elevated expression of TWIST1 with patient disease-free survival (DFS) and overall survival (OS). Elevated expression of TWIST1 was significantly associated with shorter DFS and OS in patients with type I endometrial cancer.

Kaplan–Meier analysis was used to determine the prognostic value of elevated TWIST1 expression in type I EC patients. The survival curves in Figure 2 show the association between elevated TWIST1 expression with DFS and OS in 263 type I ECs. Significance was tested in univariate and multivariate Cox regression models. The Kaplan–Meier analysis showed that high TWIST1 expression was significantly associated with shorter DFS (P = 4.6 E^−4^) and OS (P = 2.87 E^−3^). Furthermore, Cox univariate proportional hazards analysis showed that menopause, FIGO stage, differentiation, myometrial invasion, LVSI, LNM, and elevated TWIST1 expression predicted a significantly shorter DFS and OS. More importantly, multivariate analysis showed that high TWIST1 expression (HR, 2.361; 95% CI, 1.081–5.159; *P* = .03), in addition to myometrial invasion, LVSI, and LNM, were independent predictors of worse DFS in patients with type I ECs. Our data showed that LNM alone was an independent predictor of shorter OS, whereas high TWIST1 expression had no significant correlation with OS in patients with type I ECs (Table 3).

**Table 3:** Univariate and multivariate analysis of the correlation between prognostic value and disease-free survival (DFS) and overall survival (OS) in 263 patients with type I endometrial cancers.

	Disease-free survival			Overall survival		
Characteristic	HR	95% CI	*P*	HR	95% CI	*P*
Univariate analyses						
Age	1.704	0.838–3.465	.141	1.537	0.714–3.306	.272
Menopause	3.542	1.367–9.175	.009	4.081	1.419–11.741	.009
FIGO stage	2.768	1.394–5.496	.004	2.346	1.129–4.876	.022
Differentiation	3.118	1.563–6.219	.001	2.962	1.414–6.207	.004
Myometrial invasion	7.900	3.876–16.102	1.3E^−8^	7.367	3.465–15.662	2.1E^−7^
Tumor size	1.167	0.506–2.690	.717	1.339	0.571–3.141	0.502
LVSI	11.14	5.611–22.113	5.6E^−12^	10.08	4.859–20.912	5.5E^−10^
LNM	12.96	6.482–25.923	4.3E^−13^	15.948	7.630–33.335	1.8E^−13^
TWIST1 expression	4.019	1.948–8.294	1.7E^−4^	3.743	1.740–8.056	.001
Multivariate analyses						
Menopause	1.957	0.705–5.429	.197	2.114	0.687–6.504	.192
FIGO stage	0.841	0.351–2.018	.699	0.588	0.228–1.516	.272
Differentiation	1.634	0.750–3.561	.216	1.729	0.757–3.949	0.194
Myometrial invasion	2.485	1.016–6.076	.046	2.010	0.740–5.456	.171
LVSI	3.112	1.191–8.136	.021	2.438	0.869–6.842	.090
LNM	2.813	1.023–7.733	.045	5.927	1.917–18.319	.002
TWIST1 expression	2.361	1.081–5.159	.031	2.024	0.864–4.744	.105

## 4 Discussion

TWIST1 was originally identified as an essential regulator during embryogenesis, particularly in mesoderm formation, specification, and differentiation. A large number of studies have demonstrated that TWIST1 is implicated in tumor initiation, stemness, angiogenesis, dissemination, and chemoresistance, however, few reports have elaborated the independence and interdependency of the multiple distinct pathologic functions of TWIST1 in EC.^[17]^ AH is known as a pre-cancerous lesion of EC. Lacey analyzed cases of AH that progressed to EC at least 1 year following an index AH diagnosis, and demonstrated a 40% probability of developing EC following a diagnosis of AH compared to a 10% probability when atypia were not present, commented on the need to increase sensitivity and specificity when diagnosing AH and to find methods of identifying rare non-atypical endometrial hyperplasia lesions that are also likely to progress to EC.^[18]^ Several biomarkers were analyzed to predict the response to AH, however, the results have been conflicting.^[3]^

TWIST1 may be a potential diagnostic biomarker. In our study we found a gradually increased expression of TWIST1 from NE through AH, and finally to type I ECs, although there was no significant difference in TWIST1 expression in AH and type I EC tissues. There were 8 patients (29.6%) with AH and high TWIST1 expression, which is in agreement with the previous reports, Feng^[19]^ and Senol^[20]^ reported that 39.8% (11/28) and 32.7% (16/49) of AH tissues had high TWIST1 expression, respectively. It is striking that the rate of high TWIST1 expression in the current study was similar to the existing literature (30%–40%) among AH patients with concurrent carcinoma.^[6]^ Is this coincidence? May be not. Up-regulation of TWIST1 with down-regulation of ER expression has been uncovered in breast cancer,^[21]^ which is another common malignancy in women and is also estrogen-related. Han showed estrogen promotes progression of hormone-dependent breast cancer via the CCL2-CCR2 axis by up-regulation of TWIST1 and PI3K/AKT/NF-κB signaling.^[22]^ It has been well-established that an increase in the ratio of estrogen-to-progesterone is a key driver in the progression of type I EC. There is a negative correlation between TWIST1 and ER in EC, which was confirmed by Senol.^[20]^ In addition, we found that 5.5% of patients (3/55) with NE had high TWIST1 expression, we do not know if these patients have the potential to progress to EC, current studies have only hint that high expression of TWIST1 is an important marker for the transition from hyperplasia through AH to malignancy. Further studies, including molecular genetic analyses, are warranted to confirm this hypothesis.

We assessed the correlation between TWIST1 expression and the clinicopathologic features of type I EC. There was a statistically significant positive correlation between high TWIST1 expression and deep myometrial invasion, which was also confirmed by Satoru^[12]^ and Xie.^[16]^ however, initial studies found no statistically significant association between TWIST1 and pelvic lymph node metastasis (*P* > .05). The authors commented that their patients with pelvic lymph node metastases were limited (n = 5 and n = 2, respectively). We found a statistically significant relationship between high expression of TWIST1 and lymph node metastasis (n = 24, *P* = .001). In addition, we found a statistically significant relationship between high expression of TWIST1 and LVSI (n = 29, *P* = .021), which was also not mentioned in previous studies. Our results showed that those patients who expressed high TWIST1 expression had greater invasive capability and an increased risk of developing distal metastases. On the other hand, is that means TWIST1 involved in another steps of the tumor invasion and metastasis process other than the TWIST1/E-cadherin/EMT pathway^[23]^ which was confirmed in Satorus^[12]^ and Xies^[16]^ research, such as the formation of invadopodia,^[13]^ intravascular migration, extravasation^[24]^ and vasculogenic mimicry formation,^[25]^ these pathologic functions were demonstrated in vitro cell experiments, testing proteins, such as VEGFα, ITGβ1, and VE-cadherin in our samples may help us further understand TWIST1 function in the progression of EC.

Finally, our research compared the relevance of TWIST1 to existing histomorphologic and grading systems, which differs from other studies that have been conducted. We followed these patients for a long time. Based on univariate analyses, we found a positive association between TWIST1 expression and worse DFS and OS in patients with type I EC; this result was consistent with a previously published study by Satoru.^[12]^ In addition, we evaluated all the statistically significant variables using multivariate analysis, for the first time, we reported that elevated TWIST1 expression, together with deep myometrial invasion, LVSI, and LNM, was an independent predictor of shorter DFS in patients with type I EC. As mentioned above, compared with these pathologic features, testing TWIST1 is more reproducible and accurate, thus showing the potential value in molecular classification.

### 4.1 Limitations

Our study had several limitations. This was a retrospective study with a relatively small number of patients. Some biomarkers, such as ER and E-cadherin, were not included. Further research with a large sample size and molecular genetic analyses is needed. We sought to determine the level of concordance between endometrial biopsies and subsequent hysterectomy specimens for further clinical application.

## 5 Conclusion

In conclusion, our study found a gradually increased expression of TWIST1 from NE through AH, and finally to type I EC. Elevated TWIST1 expression was significantly associated with some clinical variables that indicated poor prognosis, including FIGO stage, myometrial invasion, LVSI, and LNM. More importantly, there was evidence of a statistically significant difference between high TWIST1 expression and worse prognosis in patients with type I EC; however, it may be debated whether or not the findings are clinically relevant. Additional well-designed cohort studies are needed to confirm the association.

## Author contributions

**Conceptualization:** Xiaoxia Bai, Fenfen Wang, Baohua Li.

**Data curation:** Junhua Shen, Na Li.

**Formal analysis:** Junhua Shen.

**Funding acquisition:** Fenfen Wang, Baohua Li.

**Investigation:** Junhua Shen, Na Li.

**Methodology:** Junhua Shen, Qin Chen, Baohua Li.

**Project administration:** Junhua Shen, Qin Chen, Xiaoxia Bai.

**Resources:** Qin Chen, Fenfen Wang, Baohua Li.

**Software:** Junhua Shen.

**Visualization:** Junhua Shen.

**Writing – original draft:** Junhua Shen.

**Writing – review & editing:** Junhua Shen, Baohua Li.
